# Estimation of the fraction of absorbed photosynthetically active radiation (fPAR) in maize canopies using LiDAR data and hyperspectral imagery

**DOI:** 10.1371/journal.pone.0197510

**Published:** 2018-05-29

**Authors:** Haiming Qin, Cheng Wang, Kaiguang Zhao, Xiaohuan Xi

**Affiliations:** 1 Key Laboratory of Digital Earth Science, Institute of Remote Sensing and Digital Earth, Chinese Academy of Sciences, Beijing, China; 2 University of Chinese Academy of Sciences, Beijing, China; 3 Ohio Agricultural and Research Development Center, The Ohio State University, Wooster, Ohio, United States of America; College of Agricultural Sciences, UNITED STATES

## Abstract

Accurate estimation of the fraction of absorbed photosynthetically active radiation (fPAR) for maize canopies are important for maize growth monitoring and yield estimation. The goal of this study is to explore the potential of using airborne LiDAR and hyperspectral data to better estimate maize fPAR. This study focuses on estimating maize fPAR from (1) height and coverage metrics derived from airborne LiDAR point cloud data; (2) vegetation indices derived from hyperspectral imagery; and (3) a combination of these metrics. Pearson correlation analyses were conducted to evaluate the relationships among LiDAR metrics, hyperspectral metrics, and field-measured fPAR values. Then, multiple linear regression (MLR) models were developed using these metrics. Results showed that (1) LiDAR height and coverage metrics provided good explanatory power (*i*.*e*., R^2^ = 0.81); (2) hyperspectral vegetation indices provided moderate interpretability (*i*.*e*., R^2^ = 0.50); and (3) the combination of LiDAR metrics and hyperspectral metrics improved the LiDAR model (*i*.*e*., R^2^ = 0.88). These results indicate that LiDAR model seems to offer a reliable method for estimating maize fPAR at a high spatial resolution and it can be used for farmland management. Combining LiDAR and hyperspectral metrics led to better performance of maize fPAR estimation than LiDAR or hyperspectral metrics alone, which means that maize fPAR retrieval can benefit from the complementary nature of LiDAR-detected canopy structure characteristics and hyperspectral-captured vegetation spectral information.

## Introduction

Vegetation plays an important role in the exchange of energy and matter between atmosphere and land, and it provides food and habitats for terrestrial species. The photosynthetic process of green vegetation that converts sunlight to sugars is a key component of vegetation function. Photosynthetically active radiation (PAR) is solar radiation in the spectrum of 400–700 nm that can be used by vegetation canopy in photosynthesis [1] with a unit of measurement of *μmol* · *m*^-2^ · *s*^-1^. Fraction of absorbed photosynthetically active radiation (fPAR) is the fraction of incoming solar radiation in the spectrum of 400–700 nm that is absorbed by vegetation canopy, and it is a ratio that ranges from 0 to 1 with no units [2]. fPAR is a key indicator of vegetative water, energy and carbon balance [3], and it is also an important parameter in ecosystem models, climate models, vegetation net primary productivity estimation models, and crop yield estimation models [4]. Therefore, efficient and accurate methods for fPAR mapping over large areas will be valuable to these important modeling efforts [5].

fPAR can be measured using traditional ground-based methods, e.g., canopy analysis systems or radiation sensors, such as SunScan, AccuPAR, and TRAC [6–8]. These ground-based measurement methods can be used to accurately obtain fPAR in real time, but they are time-consuming and labor-intensive, and they cannot be implemented over large areas [8, 9]. Remote sensing can overcome abovementioned shortcomings and obtain spectral information from canopy by non-destructive method [10, 11]. Optical remote sensing datasets have been widely used to estimate vegetation fPAR in many studies [12]. fPAR are often estimated based on the statistical relationships between field-measured fPAR and vegetation indices (VIs), such as normalized difference vegetation index (NDVI) [13–15], perpendicular vegetation index (PVI) [13], green normalized difference vegetation index (GNDVI) [16, 17], enhanced vegetation index (EVI) [18], and simple ratio (SR) [15]. Although optical remote sensing imagery can be used to rapidly estimate vegetation fPAR over large areas, these methods are often limited by the saturation of vegetation indices in dense vegetation [17].

LiDAR is an active remote sensing technique that can penetrate vegetation canopy and capture light transmissivity inside the canopy [19]. LiDAR can bypass the saturation effect and provide accurate information on vegetation structure [20, 21]; so, it has been used to estimate vegetation fPAR in several studies. Chasmer et al. [7] calculated the ratio of canopy return number to total return number as fractional canopy cover to estimate forest fPAR, and their results indicated that estimated fPAR was strongly correlated with hemispherical photography-derived fPAR. Luo et al. [19] found that LiDAR-derived canopy fractional cover had a strong correlation with field-measured maize fPAR by linear regression analysis, especially when LiDAR intensity correction was carried out. However, LiDAR metrics-based fPAR estimation methods are often limited by a lack of available spectral information [19].

In summary, airborne LiDAR data can provide detailed vegetation structural information, and hyperspectral imagery can offer abundant canopy spectral information. In these circumstances, combination of airborne LiDAR and hyperspectral data seems to be an interesting option for better estimation of maize fPAR. Previous studies have demonstrated the ability of using the combination of airborne LiDAR and hyperspectral data to estimate vegetation canopy parameters, such as leaf area index (LAI) [22], height [22, 23], biomass [24, 25], and even fPAR in boreal mixedwood forests [26]. However, no literature has focused on crop fPAR estimation by combining airborne LiDAR and hyperspectral data. Therefore, this study is a new attempt to estimate maize fPAR by combining airborne LiDAR and hyperspectral data.

The objective of this study is to determine if maize fPAR is best estimated using airborne LiDAR data or hyperspectral data, or the combination. More specifically, our goals are to (1) explore the relationships among field-measured maize fPAR with airborne LiDAR data derived height and coverage metrics; (2) evaluate the utility of using hyperspectral data-derived vegetation indices for maize fPAR estimation; and (3) determine whether the combination of LiDAR and hyperspectral data will improve the accuracy of maize fPAR estimation.

## Study area and data

### Study area

**Fig 1:**
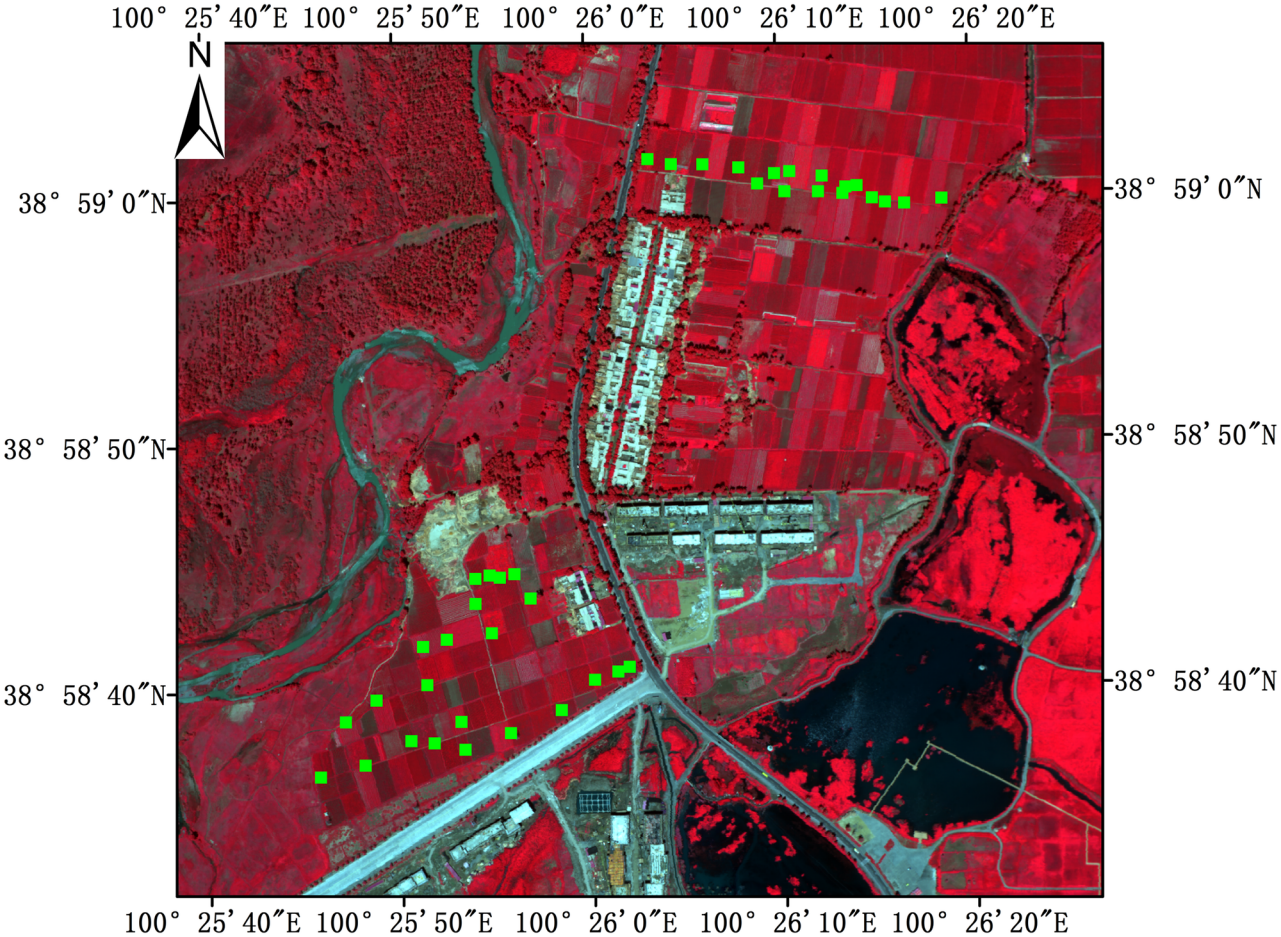
The false color composite image (NIR/red/green band) of the study area derived from the Compact Airborne Spectrographic Imager (CASI) hyperspectral image and the sampling plots (green squares in the image).

The study region is located in Zhangye City of Gansu Province in northwest China (38°58′32″-38°59′6″N, 100°25′38″-100°26′27″E). The study area is relatively flat with elevations from 1402 to 1418 m. The climate type of the study area is a temperate arid climate with an annual average temperature of 6 °C and an annual average precipitation of 198 mm [27]. Maize is the main vegetation in the study area, and it is often sowed in late April and harvested in mid-September. Fig 1 shows a false-color composite image of the study area, and the green squares are the locations of the sampling plots. The false-color image was derived from a Compact Airborne Spectrographic Imager (CASI) hyperspectral image, and the near-infrared band (826.3 nm), red band (654.8 nm), and green band (540.4 nm) are shown in red, green, and blue, respectively.

### Field measurements

The field measurements were conducted on July 8–13, 2012, when the maize was at the pre-flowering stage. In this stage, maize had almost the maximum leaves, and the top of maize tassel was about 3 cm higher than top-most leaf. We randomly selected 40 sample plots with uniformly distributed maize to measure the fPAR by using a SunScan canopy analyzer. The owners of the land gave permissions to conduct the study on these sites. The mean density of these plots was 6 maizes/m^2^ and the mean height of the 40 maize plots ranged from 1.6 m to 2.8 m. The plot size was chosen as 4 m × 4 m according to Wang et al. [28] and the locations of all sample plots are shown in Fig 1. To acquire fPAR, above-canopy upwelling PAR (PAR_au_) and downwelling PAR (PAR_ad_), and below-canopy upwelling PAR (PAR_bu_) and downwelling PAR (PAR_bd_) were all measured at each sample plot by using SunScan canopy analyzer with the *μmol* · *m*^-2^ · *s*^-1^ units of the measurement. The PAR_au_ and PAR_ad_ were measured by SunScan canopy analyzer at an approximate height of 0.5 m above canopy surface, and PAR_bu_ and PAR_bd_ were measured at an approximate height of 0.15 m above ground to reduce the impacts from short grasses. The sensor was kept horizontal during all measurements to reduce error. For each plot, fPAR was calculated based on Eq (1).

$$fPAR=\frac{\left(PAR_{ad}-PAR_{au})-(PAR_{bd}-PAR_{bu}\right)}{PAR_{ad}}$$ (1)

To cover plot and minimize error, we measured fPAR in the center and four cardinal directions at each plot and then averaged these measurements to obtain field-measured fPAR. Measurements were taken under sunny and cloudless skies over six consecutive days from 10:00 to 14:00. An RTK-GPS (global positioning system) was used to record geographic coordinates of the center of each plot.

### Airborne LiDAR data

Airborne LiDAR data for the study area were obtained using a Leica ALS70-HA system on July 19, 2012. The weather conditions were sunny and cloudless. Flight height was approximately 1300 m above ground with an approximate overlap of 60%, and flight speed was approximately 216 km/h. Pulse rate of the airborne LiDAR system was 210.7 KHz. LiDAR system emitted a laser pulse at a wavelength of 1064 nm, a scan angle of ±18°, and a beam divergence of 0.22 mrad. Acquired LiDAR data were discrete point cloud data with multiple returns and with a point density of approximately 7.4 points/m^2^. The LiDAR system recorded geographical coordinate, pulse intensity, return number, and scan angle.

### Hyperspectral data

Airborne hyperspectral data for the study area were collected on July 7, 2012 using a Compact Airborne Spectrographic Imager (CASI-1500). The weather conditions were sunny and cloudless. Flight height was approximately 2000 m above ground, and field of view was 40°. Swath width was approximately 1500 m. The CASI-1500 has 48 spectral bands over wavelengths from 382.5 nm (visible band) to 1055.5 nm (near infrared band), and the horizontal resolution of hyperspectral data was 1 m.

## Methods

### Airborne LiDAR data processing

There are some outliers in the airborne LiDAR point cloud data, so the noises were first removed based on the frequency histogram. To separate ground from non-ground points, the cloth simulation filter (CSF) algorithm [29], which is embedded in the Point Cloud Magic software (China), was used to filter the point cloud data. The filtered ground points and non-ground points were used to generate the digital terrain model (DTM) and digital surface model (DSM) with a spatial resolution of 1 m respectively.

To remove the influence of terrain, we extracted the corresponding ground elevation of each non-ground point from DTM and then subtracted it from original point height to obtain normalized height of each non-ground point. The point cloud data within each plot were extracted to calculate LiDAR metrics. Twenty height-related and coverage-related LiDAR metrics (Table 1) that have been commonly used in vegetation parameter estimations were extracted according to the metric descriptions in Table 1. The point cloud intensity was affected by incidence angle and sensor-to-target distance. To calculate intensity-based fractional cover (*fcover*_*intensity*_), the return intensity was corrected using incidence angle and distance according to Eq (2). Then, the sum of vegetation return intensity and the sum of ground return intensity within each plot were calculated. The *fcover*_*intensity*_ was calculated according to Eq (3).

$$I_{corrected}=I\cdot \frac{D^{2}}{D_{s}^{2}\text{cos}\alpha }$$ (2)

$$fcover_{intensity}=\frac{I_{canopy}}{I_{canopy}+kI_{ground}}$$ (3)

where *D* is the distance from sensor to target, *D*_*s*_ is the perpendicular distance from sensor to target, *α* is the incidence angle of laser pulse, *I* is raw echo intensity, and *I*_*corrected*_ is the echo intensity corrected by distance and incidence angle. *I*_*canopy*_ is the sum of vegetation return intensity, *I*_*ground*_ is the sum of ground return intensity, *k* is reflectance adjusting factor, which is set to 2.0 according to Luo et al. [19], and *f*cov*er*_int*ensity*_ is intensity-based fractional cover.

**Table 1:** LiDAR metrics used for maize fPAR estimation.

LiDAR metrics	Descriptions
R_veg_grd_	The ratio of vegetation return number to ground return number
fcover_intensity_	Intensity-based fractional cover, calculated as the ratio of vegetation return intensity to all return intensity
H_5th_	5^th^ percentile vegetation point cloud height
H_10th_	10^th^ percentile vegetation point cloud height
H_25th_	25^th^ percentile vegetation point cloud height
H_50th_	50^th^ percentile vegetation point cloud height
H_75th_	75^th^ percentile vegetation point cloud height
H_90th_	90^th^ percentile vegetation point cloud height
H_95th_	95^th^ percentile vegetation point cloud height
IQR_H_	Interquartile range (IQR) of vegetation point cloud height; IQR_H_ = H_75th_ − H_25th_
CV_H_	Coefficient of variation of vegetation point cloud height
Range_H_	Difference between maximum vegetation point cloud height and minimum vegetation point cloud height
Mean_H_	Mean value of vegetation point cloud height
CNR_H_	Canopy relief ratio of vegetation point cloud height; CNR_H_ = (Mean_H_ −minimum vegetation point cloud height)/ Range_H_
MAD_H_	Median absolute deviation (MAD) from median vegetation point cloud height; MAD = 1.4826*median(|height-median height|)
AAD_H_	Mean absolute deviation (AAD) from mean vegetation point cloud height; AAD = mean(|height-mean height|)
Variance_H_	Variance of vegetation point cloud height
Stdev_H_	Standard deviation of vegetation point cloud height
Skewness_H_	Skewness of vegetation point cloud height
Kurtosis_H_	Kurtosis of vegetation point cloud height

### Hyperspectral data processing

To reduce the effects of atmospheric scattering and absorption, atmospheric correction for the airborne hyperspectral image was carried out using the FLAASH (Fast Line-of-Sight Atmospheric Analysis of Spectral Hypercubes) correction module, which is embedded in ENVI software. In the FLAASH correction module, atmospheric model was mid-latitude summer, aerosol model was rural, sensor type was CASI, and sensor altitude, ground elevation, flight date and other parameters were all set according to data specifications. The DSM was used as reference image to geometrically calibrate the hyperspectral image by using image-to-image registration module, which is embedded in ENVI software. Thirty ground control points (GCPs) were selected to establish calibration model to execute geometric calibration, and root mean square (RMS) was 0.203. The hyperspectral images were then mosaicked using the mosaic function in ENVI software to obtain an image that covered entire study area. Finally, the hyperspectral data within each plot were extracted and used to calculate hyperspectral metrics.

According to previous studies, based on spectral characteristics of maize and physical meaning of spectral index, a total of 46 hyperspectral vegetation indices that were related to fPAR, leaf area index (LAI), and chlorophyll (known as an important influencing factor of fPAR) were considered (Table 2) as the independent metrics to estimate maize fPAR. These indices included simple ratio indices, normalized difference ratios, triangular vegetation indices, modified versions of these three types of indices, derivative spectral indices and red edge position-based indices.

**Table 2:** Hyperspectral metrics used for maize fPAR estimation.

Hyperspectral metrics	Symbols	Formulas
Photochemical reflectance index	PRI	(R_526_-R_569_)/(R_526_+R_569_)
Modified NDVI	mNDVI	(R_755_-R_712_)/(R_755_+R_712_)
Carter index	Ctr2	R_698_/R_755_
Carotenoid reflectance index	CRI	(1/R_512_)-(1/R_698_)
Anthocyanin reflectance index	ARI	(1/R_555_)-(1/R_698_)
Vogelmann red edge index 1	VOG1	R_741_/R_726_
Vogelmann red edge index 2	VOG2	(R_741_-R_755_)/(R_712_-R_726_)
Simple ratio 1	SR1	R_411_/R_712_
Simple ratio 2	SR2	R_411_/R_698_
Simple ratio 3	SR3	R_783_/R_769_
Simple ratio 4	SR4	R_755_/R_712_
Simple ratio 5	SR5	R_898_/R_683_
Simple ratio 6	SR6	R_798_/R_669_
Simple ratio 7	SR7	R_669_/(R_555_×R_712_)
Transformed vegetation index	TVI	0.5×[120×(R_755_-R_555_)-200×(R_669_-R_555_)]
Modified transformed vegetation index	MTVI	1.2×[1.2×(R_798_-R_555_)-2.5×(R_669_-R_555_)]
Modified chlorophyll absorption in reflectance index	MCARI	[(R_698_-R_669_)-0.2×(R_698_-R_555_)]×(R_698_/R_669_)
Optimized vegetation index 1	VIopt1	R_755_/R_726_
Optimized vegetation index 2	VIopt2	100×(lnR_755_-lnR_726_)
Pigment specific simple ratio 1	PSSR 1	R_798_/R_683_
Pigment specific simple ratio 2	PSSR 2	R_798_/R_641_
Pigment specific simple ratio 3	PSSR 3	R_798_/R_469_
Sum green index	SGI	Normalized mean reflectance of 500–600 nm
Structure intensive pigment index	SIPI	(R_798_-R_440_)/(R_798_-R_683_)
Normalized pigments chlorophyll ratio index	NPCI	(R_683_-R_426_)/(R_683_+R_426_)
Red-edge vegetation stress index	RVSI	(R_712_+R_755_)/2-R_726_
Double difference index	DDI	(R_755_-R_726_)-(R_698_-R_669_)
Difference vegetation index	DVI	R_798_-R_683_
Transformed chlorophyll absorption in reflectance index	TCARI	3×[(R_698_-R_669_)-0.2×(R_698_-R_555_)×(R_698_/R_669_)]
Visible atmospherically resistant index	VARI	(R_555_-R_683_)/(R_555_+R_683_-R_483_)
Green normalized difference vegetation index	GNDVI	(R_NIR_-R_GREEN_)/(R_NIR_+R_GREEN_)
Enhanced vegetation index	EVI	(R_798_-R_683_)/(1+R_798_+6R_683_-7.5×R_483_)
Water band index	WBI	R_898_/R_969_
Triangular vegetation index	TVI	0.5×(120×(R_655_-R_555_)-200×(R_669_-R_555_))
Soil-adjusted vegetation index	SAVI	(1.5×R_798_-R_683_)/(R_798_+R_683_+0.5)
Modified soil adjusted vegetation index	MSAVI	$\left[2{\text{R}}_{798}+1-\sqrt{{\left(2R_{798}+1\right)}^{2}-8(R_{798}-R_{669})}/2\right]$
Optimal soil adjusted vegetation index	OSAVI	(1+0.16)×(R_798_-R_669_)/(R_798_+R_669_+0.16)
Red green ratio	RGratio	R_red_/R_green_
Red edge position index	REPI	Maximum value from 690 to 740 nm
Plant senescence reflectance index	PSRI	(R_683_-R_497_)/R_755_
Ratio between MTVI and MSAVI	MTVI/MSAVI	MTVI/MSAVI
Ratio between DDI and MSAVI	DDI/MSAVI	DDI/MSAVI
Ratio between MCARI and OSAVI	MCARI/OSAVI	MCARI/OSAVI
Ratio between TCARI and OSAVI	TCARI/OSAVI	TCARI/OSAVI
Derivative chlorophyll index	DCI	δ_715_/δ_726_
Maximum 1st derivative for red-edge	δ_maxred-edge_	δ_max[680–750]_

### Maize fPAR estimation method

Pearson correlation analyses were first conducted to assess the relationships among LiDAR metrics, hyperspectral metrics, and field-measured fPAR. Multiple linear regression (MLR) models, which included LiDAR metric set and hyperspectral metric set as predictor metrics, were developed independently and in combination to estimate maize fPAR. The scatterplot matrix of determination coefficient (*R*^*2*^) was used to avoid multicollinearity due to high similarity between some variables. If two or more variables were highly correlated, the more straightforward one was usually retained [30]. Standard backward stepwise regression was performed to select the metrics for final models; predictor metrics that were left in the models were significant at the 5% level [31]. Finally, we selected the best fitting models based on the lowest Akaike information criteria (AIC) value [31]. Several studies have employed the multiple linear regression method to estimate vegetation parameters, such as forest aboveground biomass [32, 33], forest height [34], and LAI [30].

### Accuracy assessment

To assess the accuracy of maize fPAR estimation, we randomly selected 25 samples out of 40 samples to develop the multiple linear regression model and then used the remaining 15 samples to validate the model. The accuracy of maize fPAR estimation model was estimated by determination coefficient (*R*^*2*^) from Eq (4) and root mean square error (*RMSE*) from Eq (5).

$$R^{2}=1-\frac{\sum_{i=1}^{n}{\left(y_{i}-x_{i}\right)}^{2}}{\sum_{i=1}^{n}{\left(y_{i}-\bar{x}\right)}^{2}}$$ (4)

$$RMSE=\sqrt{\frac{\sum_{i=1}^{n}{\left(y_{i}-x_{i}\right)}^{2}}{n-1}}$$ (5)

where *y*_*i*_ is field-measured value of the *i*th sample, *x*_*i*_ is model-estimated value of the *i*th sample, $\bar{x}$ is the average value of all model estimated values, and *n* is sample number.

## Results

### LiDAR model generation

**Fig 2:**
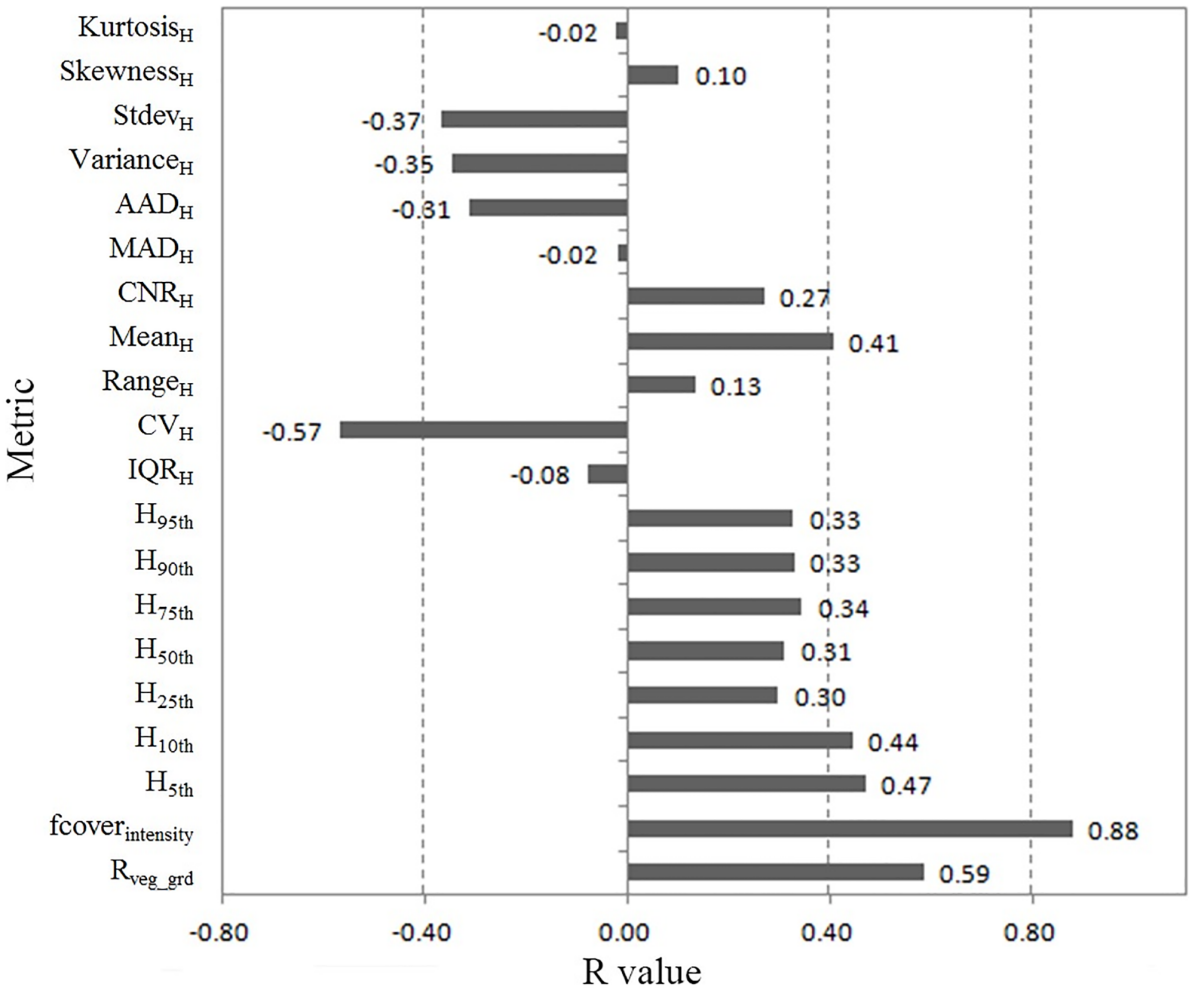
Pearson correlation coefficient (R) of each LiDAR metric and field-measured fPAR data.

To evaluate the relationship between each LiDAR metric and field-measured fPAR, a correlation analysis was conducted based on 40 field-measured fPAR data against each LiDAR metric. Pearson correlation coefficient (*R*) of each LiDAR metric and field-measured fPAR data is shown in Fig 2. Seven of the twenty LiDAR metrics had negative correlations with field-measured fPAR, and their R values were all between -0.57 and 0. Eleven of the remaining metrics had positive correlations with field-measured fPAR data, and their R values were smaller than 0.5. Two LiDAR metrics, fcover_intensity_ and R_veg_grd_, had stronger positive correlations with field-measured fPAR data, with R values of 0.88 and 0.59, respectively.

After the correlation analyses among all LiDAR predictor variables, highly inter-correlated variables were reduced, and finally 9 predictor variables were selected for model development in the end, *i*.*e*., fcover_intensity_, H_10th_, H_75th_, IQR_H_, Range_H_, CNR_H_, MAD_H_, Variance_H_, Skewness_H_. Twenty-five randomly selected samples were used to develop maize fPAR estimation model, and the remaining fifteen samples were used to validate the model. The results of multiple stepwise linear regression using LiDAR metrics are shown in Table 3. Using standard backward stepwise variable selection method, two fPAR estimation models were generated. Only fcover_intensity_ was selected as a predictor metric in the first model, while two metrics, fcover_intensity_ and CNR_H_, were selected by multiple stepwise regression method in the second model. The fcover_intensity_ is intensity-based fractional cover, which describes the status of vegetation canopy coverage. Canopy relief ratio of vegetation point cloud height (CNR_H_) describes the distribution status of vegetation point cloud, and it can illustrate maize growth status. Interestingly, height percentile metrics were not selected in automatic variable selection, and they did not provide much additional interpretation ability to fPAR estimation model.

**Table 3:** Results of the multiple stepwise linear regression using LiDAR metrics.

Model no.	Metric	R^2^	Adjusted-R^2^	RMSE	Durbin-Watson
1	fcover_intensity_	0.77	0.76	0.047	
2	fcover_intensity_ CNR_H_	0.82	0.81	0.042	1.568

According to the results shown in Table 3, the model that contained only fcover_intensity_ has an adjusted-R^2^ value of 0.76 and a RMSE of 0.047. The fPAR estimation model with both fcover_intensity_ and CNR_H_ showed a higher adjusted-R^2^ value (0.81) and a lower RMSE (0.042), so it is considered the best LiDAR based maize fPAR estimation model. Therefore, the final model generated from randomly selected modeling dataset of 25 plots is shown in Eq (6). All parameters are statistically significant at the < 0.05 level. The value of Durban-Watson test for residual autocorrelation was 1.568.

$$FPAR=0.802fcover_{\text{int}ensity}+0.268CNR_{H}-0.175$$ (6)

Using the remaining 15 samples as validation dataset, the model was run with the same predictor metrics. Validation results showed an adjusted-R^2^ value of 0.82 (p<0.05) and a RMSE of 0.040. This adjusted-R^2^ adequately corresponds to the interpretation ability of fPAR estimation model (adjusted-R^2^ = 0.81).

### Hyperspectral model generation

**Fig 3:**
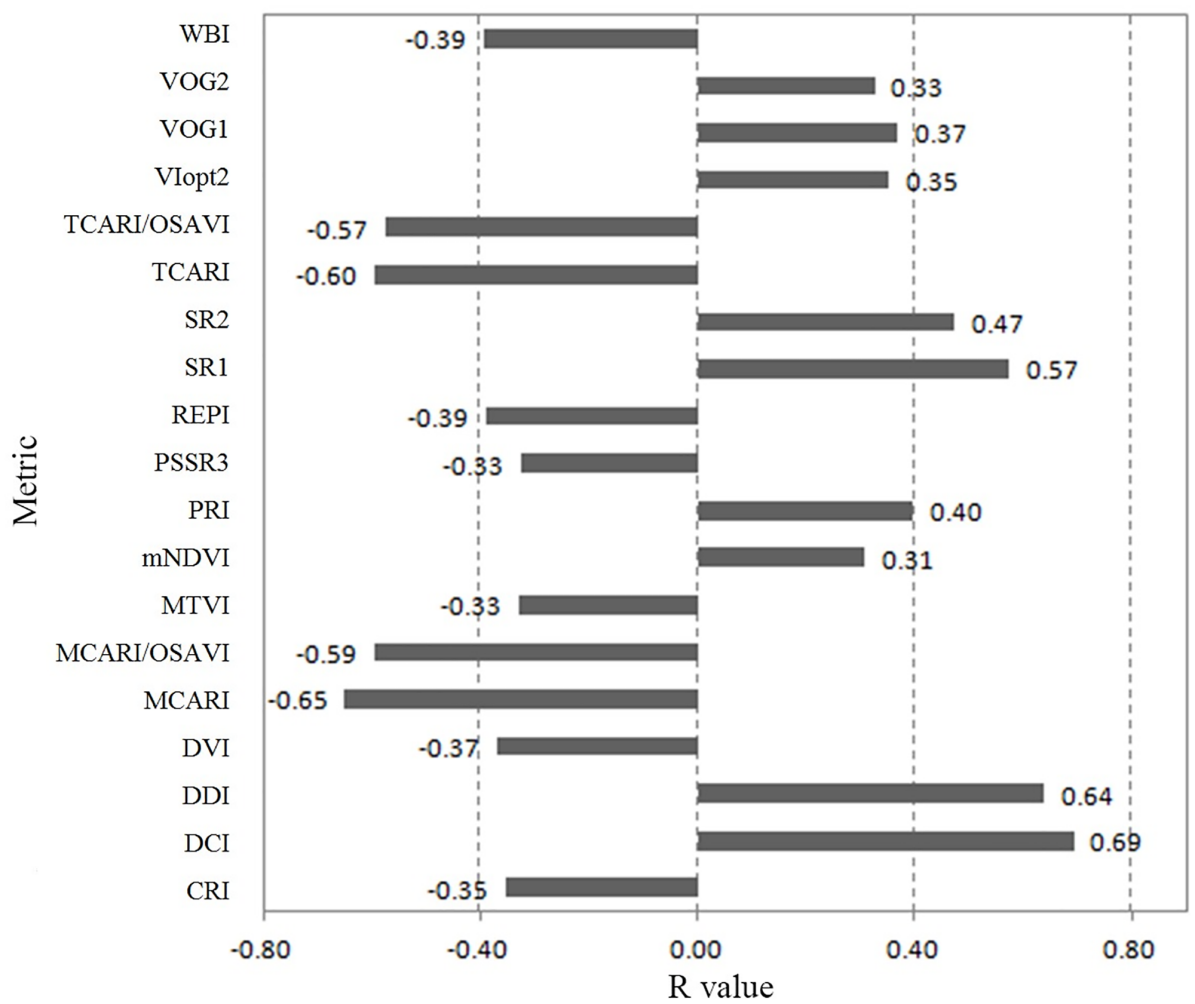
Pearson correlation coefficient (R) of each selected hyperspectral metric and field-measured fPAR.

For the 46 original hyperspectral vegetation indices, variable selection was performed using a Pearson correlation analysis. Nineteen hyperspectral metrics that had absolute R values greater than 0.3 were selected for further processing. R values of the nineteen metrics and field-measured fPAR are shown in Fig 3. Six of the nineteen hyperspectral metrics had negative correlations with field-measured fPAR with R values between -0.5 and -0.33. Four metrics, MCARI, TCARI, MCARI/OSAVI, and TCARI/OSAVI, exhibited stronger negative correlations with field-measured fPAR, with R values of -0.65, -0.60, -0.59, and -0.57, respectively. Six of the remaining metrics had positive correlations with field-measured fPAR with R values smaller than 0.5. Three hyperspectral metrics, DCI, DDI and SR1, exhibited stronger positive correlations with field-measured fPAR, with R values of 0.69, 0.64, and 0.57, respectively.

After the correlation analyses among the selected nineteen hyperspectral predictor variables, the highly inter-correlated variables were reduced, and finally ten predictor variables were selected for model development in the end, *i*.*e*., WBI, VOG2, TCRI, REPI, PRI, mNDVI, MTVI, CRI, DVI, and DCI. The twenty-five plots that were selected to generate LiDAR model were used to develop fPAR estimation model, and the remaining fifteen plots used in LiDAR model validation were used to validate the model. As the input parameters, the ten selected metrics that were derived from hyperpsectral imagery were put into the multiple stepwise regression model. Table 4 shows the results of multiple stepwise linear regression using hyperspectral metrics. After standard backward stepwise variable selection, two fPAR estimation models were generated. The first model contained only DCI, while the second model selected both DCI and VOG2 as predictor metrics. The derivative chlorophyll index (DCI) is a vegetation index related to chlorophyll content, which is the main pigment for photosynthesis by vegetation. The Vogelmann red edge index (VOG2) is sensitive to chlorophyll concentration and leaf water content and can characterize the photosynthetic capacity of vegetation. The DCI-based model has an adjusted-R^2^ value of 0.46 and a RMSE of 0.065, and the model that contained both DCI and VOG2 exhibited improved performance (adjusted-R^2^ = 0.50, and RMSE = 0.061). Therefore, in the end, maize fPAR estimation model derived from hyperspectral imagery was developed, as shown in Eq (7). The value of Durban-Watson test for residual autocorrelation is 1.232, and all parameters are statistically significant at the < 0.05 level.

FPAR=−0.813DCI−0.252VOG2+1.425 (7)

**Table 4:** Results of the multiple stepwise linear regression using hyperspectral metrics.

Model no.	Metric	R^2^	Adjusted-R^2^	RMSE	Durbin-Watson
1	DCI	0.48	0.46	0.065	
2	DCI VOG2	0.53	0.50	0.061	1.232

Using the validation dataset, the model was run with the same predictor variables. Validation results showed an adjusted-R^2^ value of 0.46 (p<0.05) and a RMSE of 0.062. This adjusted-R^2^ is also consistent with the adjusted-R^2^ value of fPAR estimation model (R^2^ = 0.50).

### Combination model generation

Further research was conducted to explore whether combining airborne LiDAR and hyperspectral data could improve the accuracy of maize fPAR estimation. Both the selected LiDAR and hyperspectral metrics were put into the multiple linear regression algorithm to develop an fPAR estimation model. As before, the modeling dataset selected to generate LiDAR model was used to develop the model, and the corresponding validation dataset was used to validate the model. Table 5 shows the results of multiple stepwise linear regression using both LiDAR and hyperspectral metrics. Three fPAR estimation models were generated after standard backward stepwise variable selection. The first and the second model were the same as LiDAR models. Three metrics, fcover_intensity_, CNR_H_, and DCI, were selected in the third model. fcover_intensity_ and CNR_H_ were both derived from LiDAR data, while fcover_intensity_ describes canopy coverage status and CNR_H_ indicates maize growth status. DCI was derived from hyperspectral data, and it represents chlorophyll content of vegetation, which is highly related to the photosynthetic capacity of maize. Finally, maize fPAR estimation model was developed, as shown in Eq (8).

$$FPAR=0.692f\text{cov}er_{\text{int}ensity}+0.251CNR_{H}-0.177DCI+0.052$$ (8)

**Table 5:** Results of the multiple stepwise linear regression using both LiDAR and hyperspectral metrics.

Model no.	Metric	R^2^	Adjusted-R^2^	RMSE	Durbin-Watson
1	fcover_intensity_	0.77	0.76	0.047	
2	fcover_intensity_ CNR_H_	0.82	0.81	0.042	
3	fcover_intensity_ CNR_H_ DCI	0.89	0.88	0.035	1.356

This model has an adjusted-R^2^ value of 0.88 and a RMSE of 0.035. All parameters are statistically significant at the <0.05 level. The Durban-Watson test for residual autocorrelation resulted in a value of 1.356.

Validation dataset was used to validate the fPAR estimation model, and the results showed an adjusted-R^2^ value of 0.89 (p<0.05) and a RMSE of 0.029, which is in accordance with the adjusted-R^2^ value of the fPAR estimation model (adjusted-R^2^ = 0.88).

## Discussion

### LiDAR model

**Fig 4:**
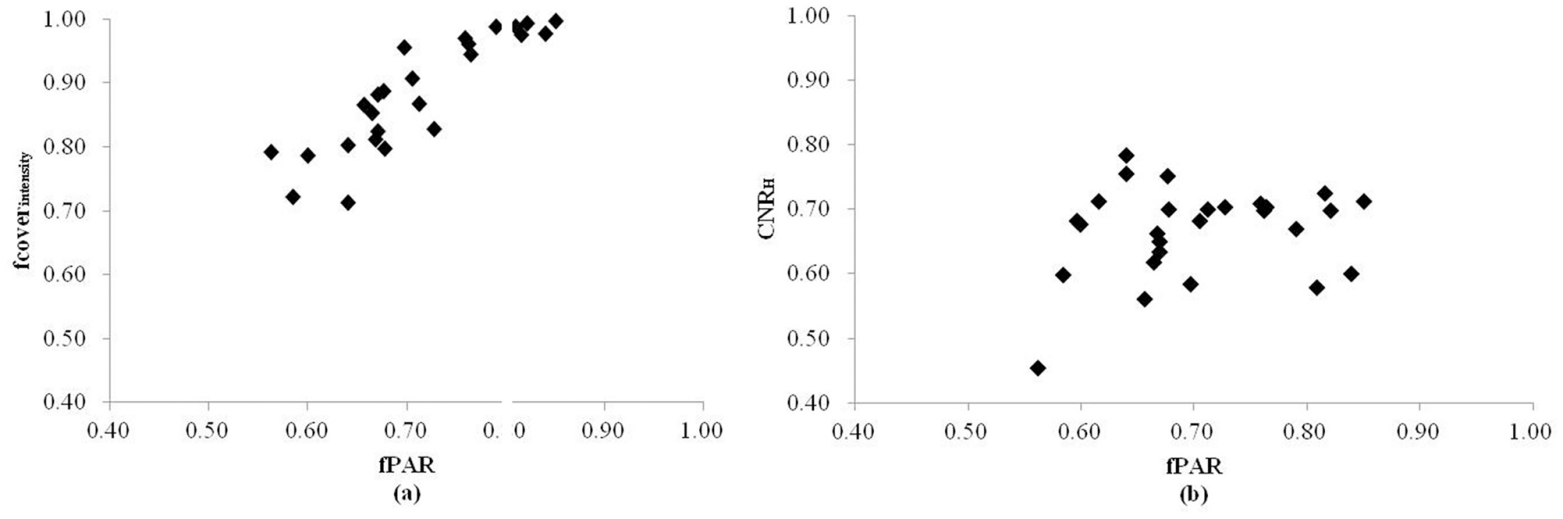
Scatterplots of fPAR and (a) fcoverintensity, and (b) CNRH from the 25 modeling plots.

The final fPAR estimation model includes two LiDAR metrics, which suggests some interesting aspects of the model and using LiDAR to predict fPAR in general. The fcover_intensity_ showed a strong and positive correlation to maize fPAR (R = 0.88), *i*.*e*., as fPAR increased, fcover_intensity_ increased (Fig 4(a)). High fcover_intensity_ values correspond to high canopy coverage and low gap fractions (*i*.*e*., low penetration). It was expected that a metric of this nature would be included in the final model since canopy coverage was high, the leaf area used for photosynthesis was large, and therefore, the fPAR was large.

Another predictor metric did not exhibit a strong correlation to maize fPAR. However, the nature of multiple linear regression algorithm is that the combination and interplay of trends between predictor variables can often generate more explanatory power than an individual variable [30]. Fig 4(b) shows relatively weak positive correlation between fPAR and CNR_H_. CNR_H_ is canopy relief ratio of vegetation point cloud height, which demonstrates the distribution status of vegetation point cloud. Higher CNR_H_ values mean that more LiDAR pulses are intercepted by upper canopy leaves, and fewer LiDAR pulses penetrate to lower canopy leaves. If canopy is dense, there is a higher chance of more pulses being intercepted by upper canopy leaves; more open canopies allow for greater pulse penetration and more returns from lower canopy leaves.

The lack of height percentile metrics captured in LiDAR model was noticeable because these metrics tend to be predominant in many other vegetation inventory variable estimation models (e.g., biomass and LAI). This phenomenon can be explained by the fact that although these height percentile metrics provide a general measure of canopy complexity and penetration, there were other metrics derived from the LiDAR data that had better explanatory abilities for maize fPAR.

**Fig 5:**
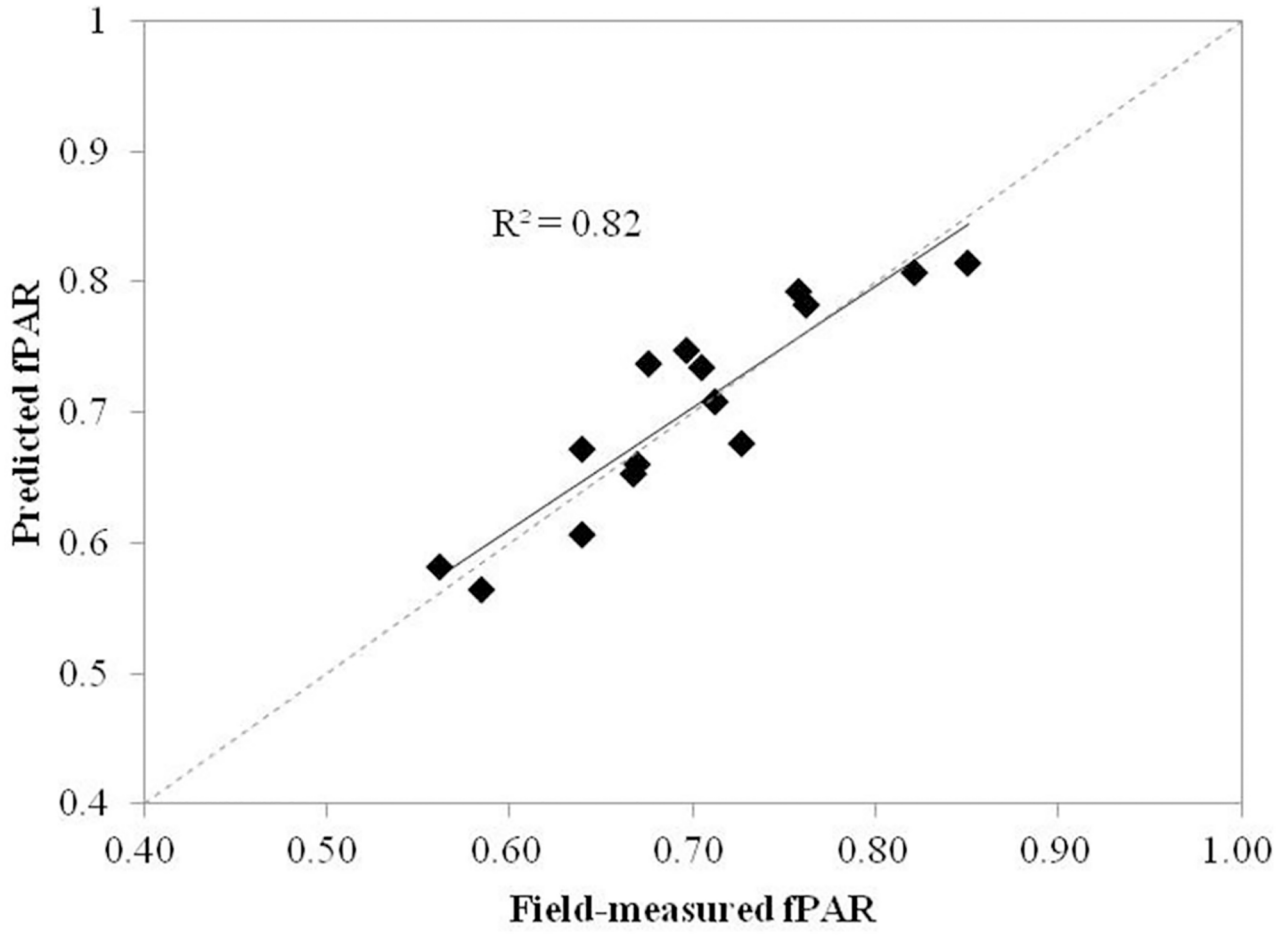
Scatterplots of field-measured fPAR values versus fPAR values predicted by the LiDAR model.

The regression model generated by the modeling dataset was used to predict maize fPAR for the validation dataset, and results are shown in Fig 5. The correlation of predicated fPAR and field-measured fPAR was strong, and the straight line fit is very close to the 1:1 line. This phenomenon explained the strong explanatory power of airborne LiDAR metrics for maize fPAR.

Several studies have estimated vegetation fPAR using only LiDAR data. Chasmer et al. [7] estimated forest fPAR using a LiDAR-derived fraction coverage, and their results indicated that LiDAR-derived fraction coverage was strongly correlated with the fPAR data derived from digital hemispherical photography (R^2^ = 0.72 and RMSE = 0.11). Luo et al. [19] used discrete-return LiDAR data-derived fraction coverage to predict maize fPAR and showed slightly higher accuracy than this study. The slight difference may be related to (1) different plot sizes, as several studies have shown that plot size has an important impact on the accuracy of vegetation parameter estimations [35–37]; (2) the use of a LI-191SA Linear Quantum PAR Sensor instead of SunScan canopy analyzer for in situ fPAR data collection, as different measurement sensors always acquire discriminatory results in the same plot because of different measurement principles; and (3) different in situ measurement times during the rapid maize growing season. Qin et al. [38] estimated maize fPAR using full-waveform LiDAR data, and their results showed that full-waveform airborne LiDAR-derived canopy fraction coverage had a slightly stronger correlation with field-measured maize fPAR than measured in this study. This difference may be caused by the abundant waveform information provided by full-waveform LiDAR data.

Similar to LiDAR data, photogrammetric data can also be used to produce 3D point clouds. However, photogrammetric data can only acquire the information of canopy top, and they almost cannot penetrate vegetation canopy. Thus photogrammetric data cannot acquire the vertical structural information of vegetation canopy, which are important for vegetation parameters estimation. Therefore, although photogrammetry-derived 3D point clouds may have higher point cloud density, they have only been used to estimate tree height with high accuracy in several studies [39, 40], and they have never been used to estimate vegetation parameters which are related to vegetation vertical structural information, such as fPAR and LAI.

### Hyperspectral model

**Fig 6:**
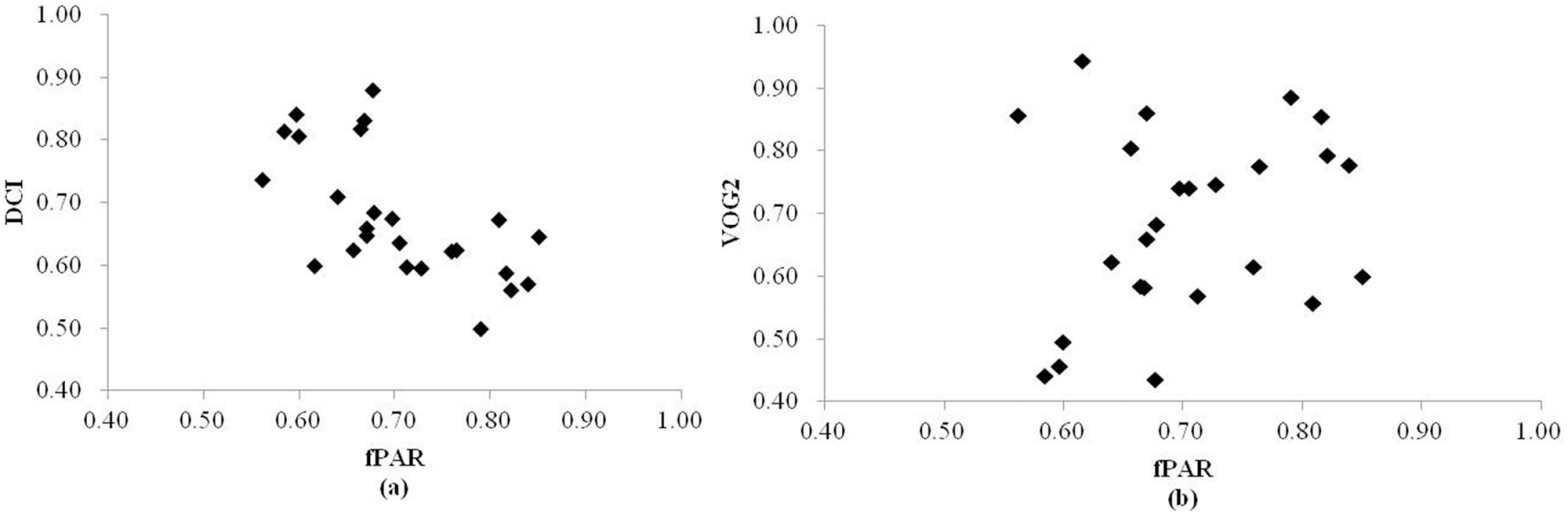
The scatterplots of fPAR and (a) DCI and (b) VOG2 from the 25 modeling plots.

Two metrics were ultimately included in the hyperspectral model. The DCI showed a moderate, negative correlation with fPAR (R = 0.69), *i*.*e*., as fPAR increased, DCI decreased (Fig 6(a)). A high DCI corresponds to low chlorophyll content. It was expected that a variable of this nature would be included in final model since chlorophyll is the main pigment utilized for vegetation photosynthesis. Chlorophyll content is a coarse surrogate for the ability of vegetation to photosynthesize (*i*.*e*., higher chlorophyll content = greater photosynthesis ability = higher fPAR).

VOG2 was another metric that was closely linked to chlorophyll content and water content. Fig 6(b) demonstrates the weak positive correlation between VOG2 and fPAR. Vegetation chlorophyll is main photosynthesis pigment in vegetation, while water is principal raw material for photosynthesis, which are both key factors in vegetation photosynthesis. In this study, the relationship between fPAR and VOG2 was weak. This phenomenon cannot be explained in this study because the relationship between VOG2 and chlorophyll content and the relationship between VOG2 and water content are not clear, which should be studied in the future.

**Fig 7:**
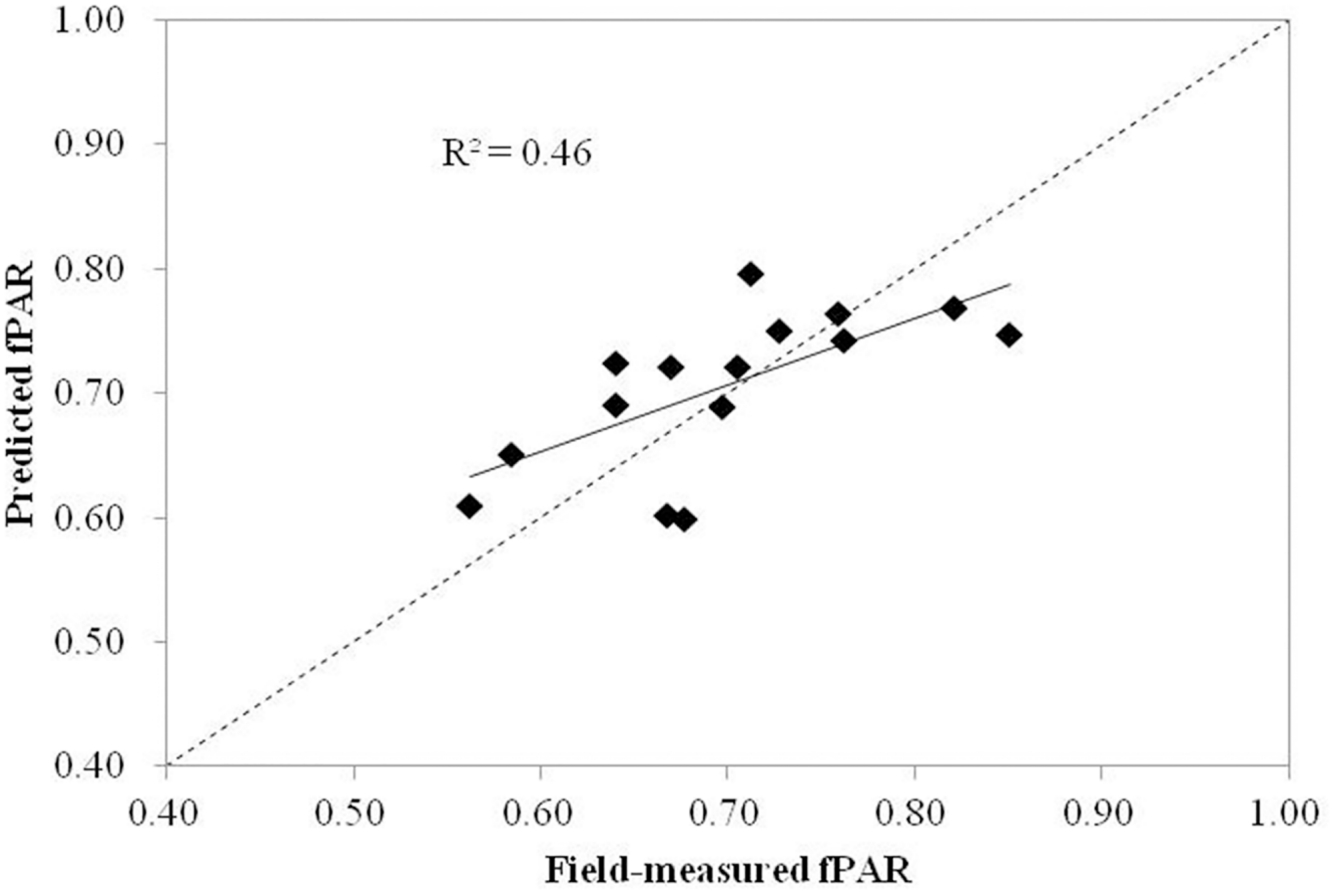
Scatterplots of field-measured fPAR versus fPAR predicted by the hyperspectral model.

The relationship between the fPAR predicted by hyperspectral model and field-measured fPAR data is shown in Fig 7. The correlation between predicated fPAR and field-measured fPAR was moderate, and the straight line fit is different from the 1:1 line. This can explain the relatively weaker explanatory power of hyperspectral metrics for maize fPAR than LiDAR metrics.

Previous studies that have used only hyperspectral data to estimate vegetation fPAR tend to show slightly better results than this study, but they used different methods. Tan et al. [17] estimated corn fPAR using hyperspectral vegetation indices, and their results showed a higher fPAR estimation accuracy (R^2^ = 0.75). This result was because a piecewise fPAR regression model—the regression variable is green normalized difference vegetation index (GNDVI) for fPAR values less than 0.75, and scaled normalized difference vegetation index (NDVI*) for fPAR values greater than 0.75 –was used to estimate corn fPAR. Yang et al. [15] used hyperspectral vegetation indices to estimate corn fPAR based on a neural network method. Although fPAR estimation accuracies were higher in these studies, the relationships between corn fPAR and vegetation indices could not be clearly acquired.

Our study showed airborne LiDAR can acquire more accurate maize fPAR than hyperspectral data, which was consistent with other vegetation parameter estimation [28, 30]. However, each method has its shortcomings in different situations, such as growing stage and terrain condition. In complicated terrain areas, hyperspectral imagery-derived canopy reflectance will be seriously distorted [41]. This phenomenon is caused by inhomogeneous incident radiation and the shadow caused by topographic occlusion, and it will affect the accuracy of vegetation parameter estimation. Similarly, LiDAR point cloud data of terrain areas always cannot be filtered accurately, which will also affect the accuracy of LiDAR metrics extraction [42]. In the early stages of vegetation growth, vegetation is sparse, so soil background produces many noises to canopy reflectance estimation [43]. When vegetation is too dense, airborne LiDAR often cannot penetrate vegetation canopy to acquire enough ground points [44]. All these situations will reduce the accuracy of vegetation parameter estimation. However, in this study, maize is in pre-flowering stage and in flat areas, so their effects on maize fPAR estimation can be ignored.

### Combination model

**Fig 8:**
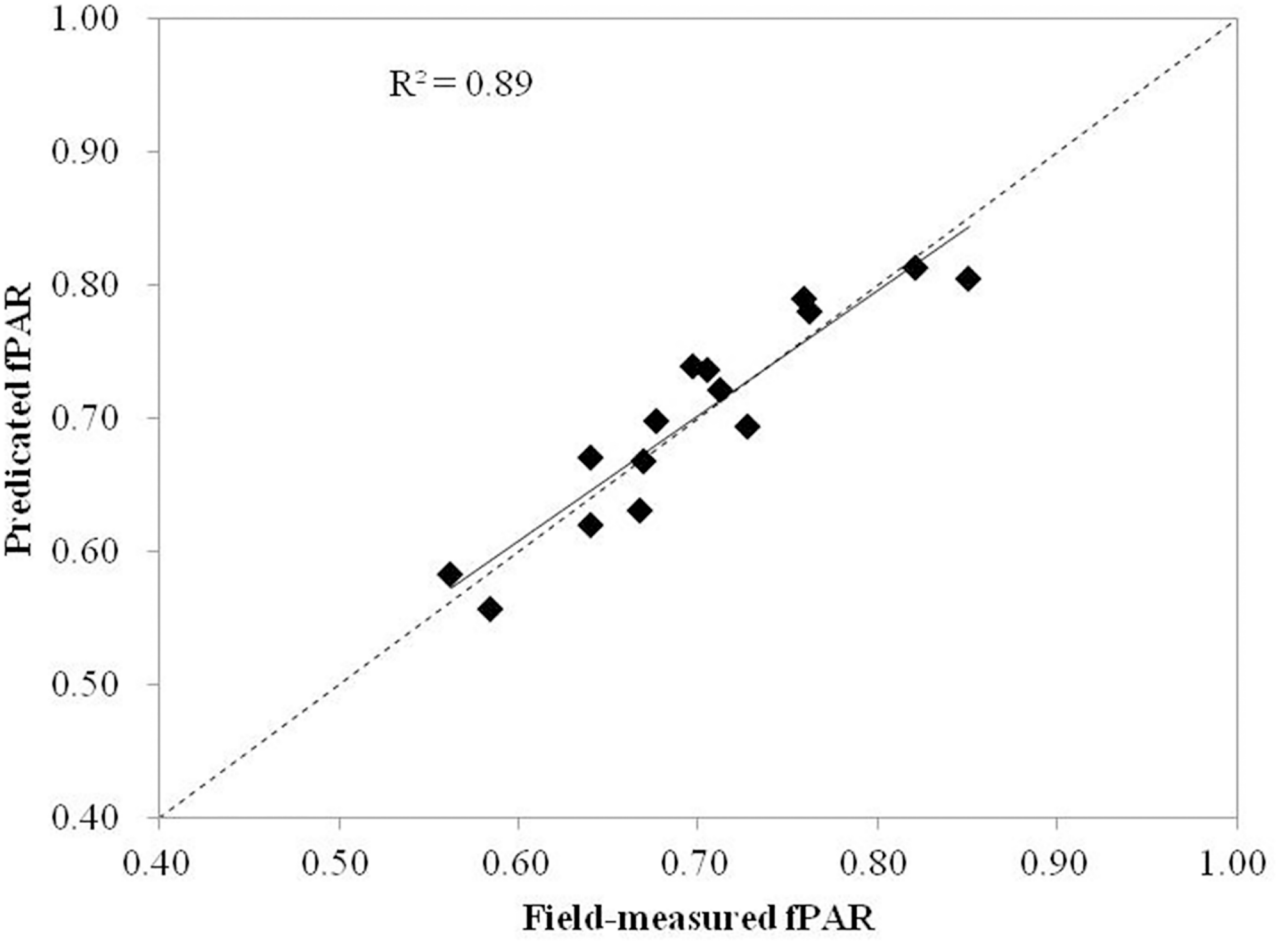
Scatterplots of field-measured fPAR values versus the fPAR values predicted from the combination model.

Two LiDAR metrics (*i*.*e*., fcover_intensity_ and CNR_H_) and one hyperspectral metric (*i*.*e*., DCI) were included in the combination maize fPAR estimation model. It was assumed that the two LiDAR metrics in conjunction with DCI derived from hyperspectral data could improve the accuracy of maize fPAR estimation to some degree (Fig 8). This result was expected considering the moderate correlation between DCI and fPAR. The combination model in Eq (8) has a moderately higher explanatory power and a moderately lower RMSE than both the LiDAR model and hyperspectral vegetation index model. This difference can be explained by the complement of LiDAR-derived canopy structure characteristics and hyperspectral-derived canopy spectral characteristics. However, the improvement to both explanatory power of the combination model and residual error is not significant considering that the combination model used three metrics, including one hyperspectral metrics (*i*.*e*., DCI), and additional expense and challenge of collecting, processing, and working with hyperspectral data.

Previous study has shown that full-waveform LiDAR data can acquire slightly higher estimation accuracy of maize fPAR than this study [38]. However, due to the limitations of the experiment conditions, cost and data storage, full-waveform LiDAR data are often unavailable. The goal of this study is to explore whether combining airborne discrete-return LiDAR data and hyperspectral data can improve the estimation accuracy of maize fPAR, and results showed that the combination model have a moderately higher accuracy than LiDAR or hyperspectral model alone. Therefore, combining airborne discrete-return LiDAR data and hyperspectral data may acquire the highest estimation accuracy for maize fPAR when full-waveform LiDAR data are not available.

Airborne LiDAR and hyperspectral data have been combined to estimate several other vegetation parameters. Wang et al. [28] found combining airborne LiDAR and hyperspectral data could provide better estimations of maize biomass. Anderson et al. [45] estimated forest basal area, above-ground biomass and quadratic mean stem diameter, and their results indicated the estimation accuracy of these parameters were improved by combining hyperspectral and LiDAR data. Thomas et al. [26] integrated airborne LiDAR and hyperspectral data to estimate boreal mixedwood forest fPAR and got an improved estimation accuracy. These studies indicated that vegetation parameters estimation could benefit from the combination of airborne LiDAR and hyperspectral data, which is in agreement with the findings of our study.

### Novelty

Several studies have estimated maize fPAR by using airborne LiDAR or hyperspectral data alone [19, 46]. However, this study is the first attempt to explore the potential of utilizing airborne LiDAR and hyperspectral data to better estimate maize fPAR, which has never been explored. The results of this study first found a moderate improvement in maize fPAR estimations when using both airborne LiDAR and hyperspectral data compared to using only airborne LiDAR or hyperspectral data. This improvement may be because airborne LiDAR data can provide detailed vegetation canopy structural information, while hyperspectral data can offer abundant spectral information about the biochemical and biophysical composition of the vegetation canopy.

### Limitations and future work

Several limitations should be identified when estimating maize fPAR using airborne LiDAR and hyperspectral data. The first limitation is the error due to the inherent discrepancies between field-measured fPAR values and airborne LiDAR and hyperspectral measurements. The measuring conditions, such as measuring distance and instrument are not consistent, which can lead to inherent errors. Another limitation is the time differences among field measurements, airborne LiDAR, and hyperspectral data acquisition. Although the time intervals are short and the weather conditions are quite similar, the small growth of maize during the days in July and the slightly different weather conditions can cause a little fPAR estimation error.

Estimating maize fPAR from different time periods during growing season may be utilized to monitor maize growth status more accurately and estimate maize yield more reliably. Due to the limitations of experimental conditions and costs, maize fPAR of only one time period was estimated in this study. However, the results of this study are effective when maize is in the pre-flowering stage. Moreover, the methods used in this study can be used to estimate maize fPAR of entire growing seasons when datasets are available.

This study took full advantage of accurate canopy structural information provided by airborne LiDAR data and abundant canopy spectral information provided by hyperspectral data to estimate maize fPAR using a multiple linear regression method. This approach was simple and efficient, and it can be applied to other sites. However, since estimation model is empirical, it could not be directly used for other vegetation types and study areas, and field-measured fPAR data are always needed to develop optimal fPAR estimation model. To develop a more accurate fPAR estimation model with wide applicability, efforts are necessary to develop a physically based fPAR estimation model that combines LiDAR and hyperspectral data in future research.

## Conclusion

This study implemented two different approaches, *i*.*e*., airborne LiDAR data and hyperspectral imagery-based approaches, to extract plot-level LiDAR and hyperspectral metrics and evaluated the capacities of LiDAR and hyperspectral metrics alone and in combination to predict maize fPAR. Results indicated that the three sets of predictive models performed well in the maize growing area, with R^2^ values from 0.50 to 0.88. LiDAR or hyperspectral metrics alone can yield fPAR estimation values with reasonable accuracies, and LiDAR model performed better than hyperspectral model. For LiDAR model, fcover_intensity_ and CNR_H_ were the selected predictors of maize fPAR, while DCI and VOG2 were selected in the final hyperspectral vegetation index model. As expected, the combination of LiDAR and hyperspectral data improved the accuracy of maize fPAR estimation to some degree, and LiDAR-derived fcover_intensity_ and CNR_H_ and hyperspectral-derived DCI predictors were included in the combination model. The improvement was attributed to the fact that fPAR is a vegetation parameter that integrates both canopy structure and spectral properties, and the data fusion approach in this study leveraged both LiDAR-derived 3D vegetation structure information and hyperspectral data-derived canopy spectral signatures. Therefore, if both LiDAR and hyperspectral data are available, the fusion of LiDAR and hyperspectral data is the best method to accurately estimate vegetation variables such as fPAR.
